# Proteasome-Generated *cis*-Spliced Peptides and Their Potential Role in CD8^+^ T Cell Tolerance

**DOI:** 10.3389/fimmu.2021.614276

**Published:** 2021-02-24

**Authors:** Artem Mansurkhodzhaev, Camila R. R. Barbosa, Michele Mishto, Juliane Liepe

**Affiliations:** ^1^Quantitative and Systems Biology, Max-Planck-Institute for Biophysical Chemistry, Göttingen, Germany; ^2^Centre for Inflammation Biology and Cancer Immunology (CIBCI) and Peter Gorer Department of Immunobiology, King's College London, London, United Kingdom; ^3^Francis Crick Institute, London, United Kingdom

**Keywords:** bioinformatics, antigen presentation, MHC-I, peptide splicing, negative selection, T-cell repertoire, T-cell tolerance

## Abstract

The human immune system relies on the capability of CD8^+^ T cells to patrol body cells, spot infected cells and eliminate them. This cytotoxic response is supposed to be limited to infected cells to avoid killing of healthy cells. To enable this, CD8^+^ T cells have T Cell Receptors (TCRs) which should discriminate between self and non-self through the recognition of antigenic peptides bound to Human Leukocyte Antigen class I (HLA-I) complexes—i.e., HLA-I immunopeptidomes—of patrolled cells. The majority of these antigenic peptides are produced by proteasomes through either peptide hydrolysis or peptide splicing. Proteasome-generated *cis*-spliced peptides derive from a given antigen, are immunogenic and frequently presented by HLA-I complexes. Theoretically, they also have a very large sequence variability, which might impinge upon our model of self/non-self discrimination and central and peripheral CD8^+^ T cell tolerance. Indeed, a large variety of *cis*-spliced epitopes might enlarge the pool of viral-human *zwitter* epitopes, i.e., peptides that may be generated with the exact same sequence from both self (human) and non-self (viral) antigens. Antigenic viral-human *zwitter* peptides may be recognized by CD8^+^ thymocytes and T cells, induce clonal deletion or other tolerance processes, thereby restraining CD8^+^ T cell response against viruses. To test this hypothesis, we computed *in silico* the theoretical frequency of *zwitter* non-spliced and *cis*-spliced epitope candidates derived from human proteome (self) and from the proteomes of a large pool of viruses (non-self). We considered their binding affinity to the representative HLA-A^*^02:01 complex, self-antigen expression in Medullary Thymic Epithelial cells (mTECs) and the relative frequency of non-spliced and *cis*-spliced peptides in HLA-I immunopeptidomes. Based on the present knowledge of proteasome-catalyzed peptide splicing and neglecting CD8^+^ TCR degeneracy, our study suggests that, despite their frequency, the portion of the *cis*-spliced peptides we investigated could only marginally impinge upon the variety of functional CD8^+^ cytotoxic T cells (CTLs) involved in anti-viral response.

## Introduction

CD8^+^ T cells are the ultimate response against viral infections. Their TCRαβ selectively recognizes viral epitope-HLA-I complexes, triggering a cytotoxic attack against infected cells in order to kill the infected cells and destroy any internal viruses. To enable this crucial immunological process, CD8^+^ TCRαβs should ideally recognize any viral (non-self) antigen to enable a robust response against viruses, and not recognize any self-antigens to avoid an autoimmune reaction resulting from cytotoxic responses directed against non-infected parenchymal cells presenting only self-antigenic peptides at their cell surface. CD8+ T cells are able to recognize a wide variety of possible non-self-antigens due to the large variety of TCRαβ variants generated during CD8^+^ T Cell maturation in the thymic cortex. Here, double negative thymocytes undergo somatic rearrangement of VDJ gene segments, causing variation in the structure and thereby binding affinities of TCRαβs expressed by different thymocytes. Through subsequent sequential positive and negative selection, only thymocytes possessing TCRαβs that do not recognize self-peptide-HLA-I complexes survive, transform into naïve CD8^+^ T cells and migrate to periphery (1). A key step of the negative selection is the recognition, by CD8^+^ TCRαβ T cell clones, of self-antigenic peptide-HLA-I complexes, which are presented by professional antigen presenting cells (APCs) in the thymic medulla. These APCs, such as mTECs and thymic Dendritic cells (DCs), express transcription factors that promote the expression of a very large variety of self-antigens, thereby promoting the identification of potentially autoreactive CD8^+^ TCRαβ T cell clones and their elimination (2). Nonetheless, thymic deletion of self-reactive CD8^+^ T cells is not perfect and many potentially autoreactive CD8^+^ T cells are present in periphery (3–6). There, they can be controlled by peripheral tolerance mechanisms such as quiescence, ignorance, anergy, and tolerance-induced cell death (5). If some of the self-epitopes recognized by potentially autoreactive CD8^+^ T cells are identical to non-self-epitopes which could be generated from viral antigens, we would expect an impaired CD8^+^ T cell response against viruses, since these potentially autoreactive CD8^+^ T cell clones would have been eliminated in the thymus or pruned in periphery.

We recently named these troubling peptides, *zwitter* epitopes (7). *Zwitter* is the German word for “hybrid,” “hermaphrodites,” originating from *zwi*-, meaning “duplex.” For example, in chemistry, a zwitterion is an ion which possesses both positively- and negatively-charged groups.

If CD8^+^ T cells specific for *zwitter* epitopes were eliminated in the thymus, they could not recognize the virus-derived *zwitter* epitope during an infection, which could create “holes” in the T cell repertoire. Likewise, if the inefficient stimulation of naïve CD8^+^ T cells or the excessive and persistent stimulation of CD8^+^ effector T cells mediated by self-derived *zwitter* epitopes induced anergy, exhaustion or peripheral deletional tolerance, these CD8^+^ T cells would be eliminated and therefore unable to recognize the virus-derived *zwitter* epitopes and to tackle a second infection.

For example, a non-synonymous mutation in a Hepatitis C Virus (HCV), which did not affect peptide-HLA-A^*^02:01 binding affinity, hampered the immune response against HCV. Since this phenomenon seemed to derive from the lack of CD8^+^ T cells with TCRαβ recognizing the mutated peptide, Wölfl et al. (8) hypothesized that HCV exploited a “hole” in the T cell repertoire. Similarly, in mouse models of vaccinia infection, ~ one-half of the vaccinia-derived epitope candidates predicted to bind Major Histocompatibility Complexes class I (MHC-I) molecules and ~ 20% of the vaccinia-derived epitope candidates identified in MHC-I immunopeptidomes by mass spectrometry (MS) did not trigger a detectable CTL response in vaccinia-immunized mice (9, 10).

Previous studies have investigated whether *zwitter* epitopes could contribute to these “holes” in the T cell repertoire by computing the overlaps between self and non-self-antigens in terms of canonical non-spliced peptide sequences (11–16). Calis et al. (17) computed that just 0.15% of all theoretical 9 amino acid long (9mer) canonical peptides derived from hundreds of viral strains completely overlap with 9mer peptide sequences present in the human proteome. Likely, this ~0.15% frequency of virus-human *zwitter* non-spliced epitopes is not sufficient to justify the hypothesized size of “holes” in the CD8^+^ TCRαβ T cell repertoire. Calis et al. (17) suggested that these “holes” could arise from the degeneracy of CD8^+^ TCRαβ specificity, as this could lead to cross-recognition of multiple antigenic peptides, thereby increasing the immunological overlap between self and non-self-antigens. However, the immunological relevance of CD8^+^ TCRαβ cross-reactivity is still a matter of debate (18–20), and even largely overlapping viral epitopes can induce an independent and non-cross-reactive T cell response (21).

**Figure 1:**
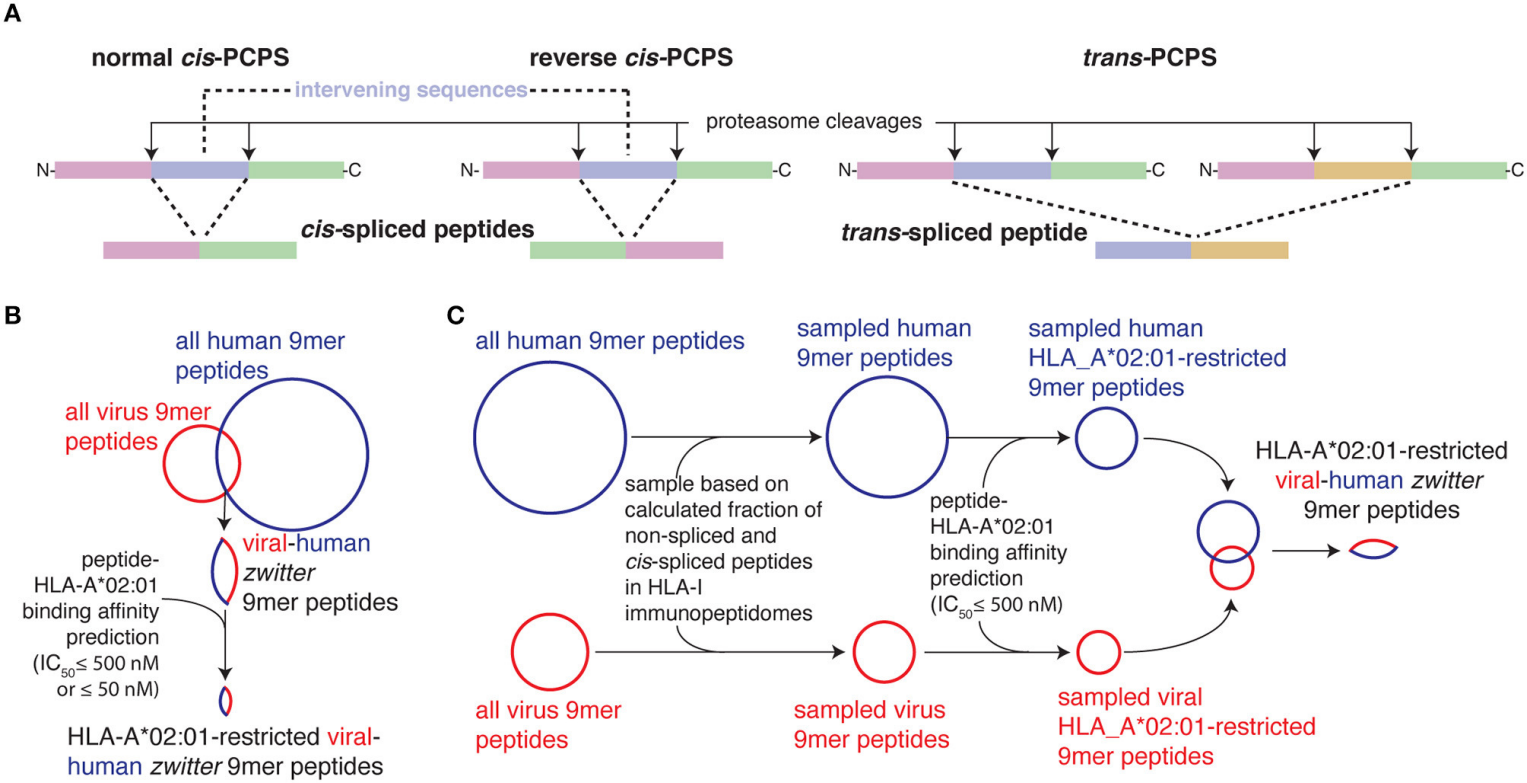
Proteasome-generated spliced peptides and *in silico* pipelines. **(A)** Proteasome-generated spliced peptides can be formed by: (i). *cis-*PCPS, when the two splice-reactants, i.e., the non-contiguous peptide fragments ligated by proteasomes, derive from the same polypeptide molecule; the ligation can occur in normal order, i.e., following the orientation from N- to C-terminus of the parental protein (normal *cis*-PCPS), or in the reverse order (reverse *cis*-PCPS); (ii). *trans*-PCPS, when the two splice-reactants originate from two distinct protein molecules or two distinct proteins. **(B,C)** *In silico* pipelines to estimate the frequency of *zwitter* epitope candidates predicted to bind HLA-A*02:01 complexes not accounting **(B)** or accounting **(C)** for non-spliced and *cis*-spliced peptide frequency in HLA-I immunopeptidomes.

Alternatively, we can consider what APCs present rather than how CD8^+^ TCRαβs recognizes epitope-HLA-I complexes on APCs. For instance, the research in this field has so far only considered canonical “non-spliced” peptides and neglected non-canonical spliced peptides bound to HLA-I complexes. Both spliced and non-spliced peptides presented to CD8^+^ T cells are mainly produced by proteasomes. These proteases can cleave antigens and release non-spliced peptides as well as ligate non-contiguous peptide fragments, thereby producing spliced peptides (22). Proteasome-catalyzed peptide splicing (PCPS) can occur by combining non-contiguous peptide fragments of the same molecule—*cis*-PCPS—or of two distinct proteins—*trans*-PCPS (Figure 1A). *Cis*-spliced peptides are produced and presented by various cells (22). They can target CD8^+^ T cell responses against otherwise neglected bacterial antigens *in vivo* in a mouse model of *Listeria monocytogenes* infection (23). They can also activate CD8^+^ T cells specific for *Listeria monocytogenes* or HIV through cross-recognition *in vivo* (24, 25). They can be neoepitopes and present recurrent driver mutations such as KRAS G12V at the cell surface of cancer cell lines (26). While, *cis*-spliced epitopes derived from melanoma-associated antigens are recognized by CD8^+^ T cells in peripheral blood of melanoma patients (27, 28). A melanoma patient with metastasis was cured through adoptive T cell therapy using an autologous tumor-infiltrating lymphocyte clone, which was proved, in a later study, to be specific for a *cis*-spliced epitope rather than any non-spliced peptides derived from the melanoma-associated antigen (29, 30).

*Cis*-spliced peptide identification is quite challenging. Estimation of their frequency in HLA-I immunopeptidomes varies from 1 to 34%, depending on the method used for their identification (31). While, although *trans*-spliced peptides have been identified in *in vitro* (26, 32–34), *in cellulo* (35) and in HLA-I immunopeptidomes (36), their immunological relevance still needs to be investigated (7).

Nevertheless, since the theoretical size of the human *cis*-spliced peptide database is extremely vast, they could make up a significant portion of the viral-human *zwitter* epitope pool and, thereby, play a role in CD8^+^ T cell tolerance. To test this hypothesis, we here computed the frequency of *zwitter cis-*spliced and non-spiced epitope candidates through comparison of human and viral proteomes. We accounted for these *zwitter* candidates' binding affinity to the most predominant HLA-I allele in Caucasian population, HLA-A^*^02:01, their estimated expression in human mTECs and their frequency in HLA-I immunopeptidomes, to accommodate these factors' potential impact on *zwitter* candidates' involvement in central tolerance.

## Materials and Methods

### Statistical Analysis

Significant difference between groups was computed by applying the Kolmogorov-Smirnov test. A *p* < 0.05 was considered statistically significant. The effect size of 9mer non-*zwitter* vs. *zwitter* peptides in binding HLA-A^*^02:01 complexes was computed via odds ratio and significance was tested using Fisher exact test, or alternatively chi square test if the sample size was too large for Fisher exact test to test significance of association. Test for association between virus length and number of *zwitter* peptides was based on Pearson's product moment correlation coefficient. Statistical values are reported in Supplementary Table 1.

In this study, we defined viral-human *zwitter* non-spiced peptides as all those non-spliced peptides from viral proteomes that completely overlapped with human non-spliced peptides. Viral-human *zwitter cis-*spliced peptides, on the contrary, included the following categories of peptides that completely overlapped between each other: viral *cis-*spliced with human non-spliced peptides, viral *cis-*spliced with human *cis*-spliced peptides, and viral non-spliced with human *cis*-spliced peptides.

### Peptide-HLA-A^*^02:01 Binding Affinity Prediction

Binding of non-spliced and *cis*-spliced 9mers to HLA-A^*^02:01 molecules was predicted using Stabilized Matrix Method (SMM) (37). This predictor showed good performance in the prediction of the binding affinity to a hundred *cis*-spliced peptides in a previous study (38). The standalone version of prediction tool was downloaded from the IEDB Analysis Resource (39). As cut-off for peptide-HLA-A^*^02:01 binding affinity we set an IC_50_ ≤ 500 nM.

In order to assess whether *zwitter* 9mer peptides were more likely to be HLA-A^*^02:01 binders than non-*zwitter* 9mer peptides on a per virus basis, we separately counted the number of non-*zwitter* and *zwitter* 9mer peptides predicted to be either non-binders or binders. Based on this contingency table the odds ratios for each virus were computed.

### Estimation of Viral-Human *zwitter* Peptides

Viral proteomes were obtained via ViralZone and the Human proteome referred to Swiss-Prot Version 2016 excluding protein isoforms (40, 41). Only viruses with human trophism were included in any downstream analysis presented here (*n* = 109; Supplementary Table 2). The Human proteome database contained 20,191 protein entries with a total of 11,323,862 amino acid residues.

We focused our study on 9mer peptides since they represent the majority of non-spliced and *cis*-spliced peptides in HLA-I immunopeptidomes (36, 38, 42). Furthermore, we focused our study on HLA-A^*^02:01 variant since it is likely the most studied HLA-I variant and is the predominant HLA-I allele in Caucasian population.

We defined viral-human *zwitter* 9mer peptides as any 9mer peptide that had a sequence that could be obtained by either peptide hydrolysis or *cis*-peptide splicing both from self-proteins and from viral proteins.

For viral and human proteomes, we first computed all possible 9mer sequences of non-spliced peptides by cutting proteins into fragments of length nine amino acids; normal and reverse *cis*-spliced peptide sequences were computed by combining splice-reactants of any length such that the resulting *cis*-spliced peptide sequence had a length of nine amino acids and by imposing a maximal intervening sequence length ≤25 amino acids (Figure 1A), as previously described (42). Afterwards, an alignment was performed between all resulting virus and human derived peptides. We considered two peptides as identical, i.e., as viral-human *zwitter* peptides, if all of their nine amino acid residues were exactly matching. The relative frequency of viral-human *zwitter* peptides (*Fv*) was calculated as:

$$\begin{array}{l} F_{v}=100\;\frac{z_{v}}{p_{v}}, \end{array}$$

where *z*_*v*_ is the number of all viral-human *zwitter* peptides of a given virus *v*; and *p*_*v*_ is the number of all possible unique 9mer peptides derived from virus *v*. The number of viral-human zwitter peptides, z, can be computed for the comparison of non-spliced peptides only (*z*_*v,i*_), of *cis*-spliced peptides only (*z*_*v,j*_), of non-spliced viral peptides compared to *cis*-spliced human peptides (*z*_*v,k*_), and of *cis*-spliced viral peptides compared to non-spliced human peptides (*z*_*v,l*_). In our analysis, we depicted either the relative frequency of viral-human non-spliced *zwitter* peptides (*F*_*v,i*_), viral-human *cis*-spliced *zwitter* peptides (*F*_*v,cis*_) or of all (non-spliced and *cis*-spliced) viral-human *zwitter* peptides (*F*_*v,all*_). The latter was obtained via:

$$\begin{array}{l} F_{v,all}=100\frac{\left\{z_{v,i},z_{v,j},z_{v,k},z_{v,l}\right\}}{\left\{p_{v,\text{all}}\right\}}, \end{array}$$

Where {} denotes the unique set of peptide sequences and *p*_*v,all*_ are all unique non-spliced and *cis*-spliced peptides derived from virus *v*.

The above-described analysis was done based on all theoretical possible non-spliced and *cis*-spliced peptides. Next, we repeated the estimation of viral-human zwitter peptide frequency by restricting the analysis to human- and virus-derived non-spliced and *cis*-spliced peptides that efficiently bind to the HLA-A^*^02:01 molecule, i.e., to peptides that have a predicted IC_50_ ≤ 500 nM, resulting in:

$$\begin{array}{l} B_{v}=100\frac{z_{b}}{b_{v}}, \end{array}$$

where *B*_*v*_ is the frequency of viral-human *zwitter* peptide restricted to HLA-A^*^02:01, *z*_*v,b*_ is the number of all viral-human *zwitter* peptides of a given virus *v* that bind HLA-A^*^02:01 and *b*_*v*_ is the number of all possible unique 9mer epitope candidates derived from virus *v* that are predicted to bind HLA-A^*^02:01 with an IC_50_ ≤ 500 nM.

### Estimation of Viral-Human *zwitter* Epitope Candidates Considering the Potential Antigen Repertoire of Human mTECs

To determine the potential antigen repertoire of human mTECs, we analyzed two transcriptome databases: (i) microarray gene expression values of human mTECs (43), and (ii) single-cell RNA sequencing of TECs in human embryos (44). Although mRNA expression does not perfectly mimic HLA-I immunopeptidomes (45), it was shown to be one of the strongest factors correlated with HLA-I immunopeptidomes (46).

In (43), the material was derived from patients that underwent corrective cardiac surgery. Here, we calculated average gene expression values (reported as log_2_ transformed fluorescence intensities) across technical replicates of each mTEC subset obtained with differing versions of microarrays provided in the dataset, and took the maximum average value.

In (44), the material was derived from healthy human fetuses as a result of medically interrupted pregnancy at weeks 8, 9, and 10. We used the subset of data that ostensibly corresponded to TECs with progenitor property of mTECs (based on the expression of the mTEC markers CLDN4 and JAG1).

We performed log-normalization of gene expression values of individual cells—reported as copy number of transcripts per individual gene—number of distinct unique molecular identifiers (*UMI*)—to mitigate the relationship between sequencing depth and gene expression. We then took an average normalized gene expression value between individual cells (47, 48):

$$\begin{array}{l} x_{i}=\log\left(\frac{{100000*UMI}_{ij}}{\underset{i}{\sum }{UMI}_{ij}}+1\right), \end{array}$$

where *x*_*i*_ is the log-normalized expression of gene *i*, *UMI*_*ij*_ is the expression value of gene *i* in cell *j* prior to normalization expressed as UMI counts, and *UMI*_*j*_ is the sum of UMI counts per cell *j*.

Afterward, we defined a crude model for antigen presentation based on the gene expression values. We assumed that the chance of an antigen being presented in mTECs' HLA-I immunopeptidomes was directly correlated with the gene expression of that antigen. The limitation of this assumption is discussed above.

We first scaled and normalized the gene expression values of the processed data obtaining weights for each antigen (*w*_*i*_):

$$w_{i}=\frac{\left(E_{i}-\min\left(E\right))/(\max\left(E\right)-\min\left(E\right)\right)}{\sum_{i=1}^{n}\left(E_{i}-\min\left(E\right))/(\max\left(E\right)-\min\left(E\right)\right)},$$

where *E*_*i*_ is the expression value of gene *i* prior to normalization, and *min(E)* and *max(E)* are the minimum and maximum gene expression values in the dataset, respectively. We next sampled from the pre-computed pool of viral-human *zwitter* peptides a subset of peptides based on the weights (*w*_*i*_) of the human antigen (*i*), which the respective *zwitter* peptide was derived from. The sampling size was set at 100% of the total number of *zwitter* peptides to reflect the odds of presentation of each given peptide. Sampling was performed with replacement based on the calculated probabilities 60 times. Finally, the frequency of viral-human *zwitter* peptides considering potential antigen repertoire of mTECs compared to all viral 9mer peptides (*M*_*v*_) was computed as:

$$\begin{array}{l} M_{v}=100\;\frac{z_{m,v}}{p_{v}}, \end{array}$$

where *z*_*m,v*_ is the number of sampled viral-human *zwitter* peptides with weights *w*_*i*_ and *p*_*v*_ is the number of all possible 9mer peptides of virus *v*. Similarly, when we considered both predicted peptide-HLA-A^*^02:01 binding affinity and potential antigen repertoire of mTECs, the viral-human *zwitter* peptide frequency (*MB*_*v*_) was computed as:

$$\begin{array}{l} {MB}_{v}=100\;\frac{z_{mb,v}}{b_{v}}, \end{array}$$

where *z*_*mb,v*_ is the number of sampled viral-human *zwitter* peptides restricted to HLA-A^*^02:01 binding with weights *w*_*i*_, and *b*_*v*_ is the number of all possible 9mer peptides restricted to HLA-A^*^02:01 binding of virus *v*.

### Estimation of the Frequency of Viral-Human *zwitter* Epitope Candidates Weighing up PCPS Frequency

Not all 9mer non-spliced and *cis*-spliced peptides that could derive from the human proteome are in reality produced by proteasomes and presented through HLA-I antigen processing and presentation (APP) pathway (22). Therefore, we implemented this factor in our *in silico* analysis of *zwitter* peptides. We aimed to determine the fractions of non-spliced (*f*_*non*_) and *cis*-spliced peptides (*f*_*cis*_) produced and presented in HLA-I immunopeptidomes relative to all theoretically possible sequences:

$$\begin{array}{l} f_{non}=\;\frac{n_{non}}{N_{non}},\\ f_{cis}=\;\frac{n_{cis}}{N_{cis}}, \end{array}$$

where *n*_*non*_ and *n*_*cis*_ is the number of presented non-spliced and *cis*-spliced peptides, respectively, and *N*_*non*_ and *N*_*cis*_ is the number of all theoretically possible non-spliced and *cis*-spliced peptides, respectively, derived from a given antigen.

An estimate of *f*_*non*_ can be directly obtained from *in vitro* digestions of synthetic polypeptides with purified proteasomes. For this dataset, we used the peptide product database derived from 4 h digestions of 47 synthetic polypeptides with purified 20S standard proteasomes (34). This large database contains 2,429 unique non-spliced and 2,379 unique *cis*-spliced peptide products, which passed several quality control steps (34). We calculated the fraction of all produced 9mer non-spliced peptides (included in Specht's database) relative to all theoretically possible 9mer non-spliced peptides for each synthetic polypeptide substrate in the database. Then, we took the median value between all polypeptides as estimation of the fraction of non-spliced 9mer peptides generated by proteasomes. These calculations resulted in *f*_*non*_ ~ 0.27, i.e., ~27% of all possible non-spliced 9mer peptides are generated *in vitro* by proteasomes and detected through MS. Therefore, in the following analysis, we randomly sampled 27% of all theoretical 9mer non-spliced peptides to recompute the number of viral-human *zwitter* peptides in absence of reliable proteasome peptide hydrolysis and peptide *cis*-splicing predictors.

We could have used the same strategy to compute the fraction of *cis*-spliced peptides produced by proteasomes compared to all theoretical *cis*-spliced peptide products. However, *cis*-spliced peptides have been proved to be produced in significantly lower amount than non-spliced peptides (26, 33, 34). Bearing this in mind, we speculated that a large number of *cis*-spliced peptides produced by proteasomes *in vitro* could not pass all APP steps and become antigenic as compared to non-spliced peptides.

On the contrary, HLA-I immunopeptidomes should be more informative in such a matter, since the APP pathway should already have filtered out many *cis*-spliced peptides generated in low amount. Therefore, we used the information available about *cis*-spliced peptide frequency in HLA-I immunopeptidomes measured through MS and combined with the information of non-spliced peptide frequency in *in vitro* digestions (*f*_*non*_). Indeed, the estimation of *f*_*cis*_ based on *cis*-spliced peptide product frequency *in vitro* digestions as measured through MS could have resulted in an overestimation of *f*_*cis*_. Therefore, we defined the relative frequencies of *cis*-spliced peptides in HLA-I immunopeptidomes (*f*) as measured by MS as:

$$\begin{array}{l} f=\frac{{100\;n}_{cis}}{n_{cis}+n_{non}}, \end{array}$$

where *n*_*cis*_ is the number of *cis*-spliced peptides detected in HLA-I immunopeptidomes and *n*_*non*_ is the number of non-spliced peptides detected in HLA-I immunopeptidomes. Since *f* was estimated to be in the range of 1–34% (31). For a given estimate of *f* we could then compute the number of *cis*-spliced peptides presented in HLA-I immunopeptidomes (*n*_cis_) as:

$$\begin{array}{l} n_{cis}=\frac{{f\;N}_{non}f_{non}}{100-f}. \end{array}$$

Furthermore, we could compute the total number of all theoretical *cis*-spliced peptides (*N*_*cis*_) as:

$$\begin{array}{l} N_{cis}={\gamma N}_{non}, \end{array}$$

where γ was estimate to have a value of 398 for proteins of length 500 amino acids or longer (42). This resulted in:

$$\begin{array}{l} f_{cis}=\frac{f{\;f}_{non}}{\gamma \left(100-f\right)}. \end{array}$$

We used a range of potential frequencies of observed *cis* spliced peptides relative to the whole HLA-I immunopeptidome *f* (1–35%) to determine a range of *f*_*cis*_. Based on *f*_*cis*_ and *f*_*non*_, we randomly sampled non-spliced and *cis* spliced peptides 600 times from all viral and human proteomes without replacement. For each of the 600 samples for each *f*_*cis*_, we counted the number of all sampled HLA-A^*^02:01-restricted *zwitter* peptides.

### HIV-Derived HLA-A^*^02:01-Restricted Non-immunogenic 9mer Peptides

As proof of principle, we selected a pool of HIV-derived HLA-A^*^02:01-restricted 9mer peptides, which were previously suggested to be non-immunogenic. This pool included non-spliced epitope candidates derived from HIV, which:

(i) were investigated by Perez et al. (49) through IFN-γ ELIspot assay in HIV- infected donor peripheral blood mononuclear cells (PBMCs) pulsed/non-pulsed with synthetic epitope candidates. We considered as non-immunogenic those peptides that did not induce immune response after peptide stimulation.

(ii) were included in a database by Ogishi and Yotsuyanagi (50). This database collected outcomes of various T cell activation assays on HLA-I-restricted non-spliced peptide sequences (8–11 mer peptides). In this database, we selected HIV-derived HLA-A^*^02:01-restricted 9mer peptides, which were confirmed as non-immunogenic among all studies considered in the database.

(iii) were included in the EPIMHC database (51), which collected datasets of T cell response against epitope candidates. In this database, non-immunogenic peptides were selected by applying the following parameters: Allele, HLA A0201; Length, 9mer; MHC source, Human; Peptide source organism, HIV1; Peptide Binding Level, all; T-cell activity, all; Immunogenicity level, all; Processing, all.

The pool of peptide candidates derived from these three databases were then analyzed for peptide-HLA-I bind affinity prediction—as described above—and only peptides with predicted peptide-HLA-A^*^02:01 IC_50_ ≤ 500 nM were selected (Table 1).

**Table 1:** List of HIV-derived HLA-A^*^02:01-restricted 9mer peptides not immunogenic and their *zwitter* peptide pair.

**Peptide**	**IC_**50**_ (nM)**	**Rank**	**References**	***cis*-spliced, 25 int. seq**.	**Any *cis*-spliced**
WLWYIKIFI	24.1	0.5	(51)		
MLQLTVWGI	34.8	0.6	(49)		
LTFGWCFEL	43.5	0.8	(49)		
SITNWLWYI	44.9	0.8	(49)		
LLNATAIAV	50.7	0.9	(51)		Q9P273|TEN3_HUMAN. 1504-1506/1405-1410
QLAEVVQKV	50.5	0.9	(49)	Q14764|MVP_HUMAN.786-790/762-765	Q9NRD9|DUOX1_HUMAN. 895-900/687-689
ALQDSGLEV	56.3	1.1	(49)		Q13263|TIF1B_HUMAN. 655-658/601-605
ALQDSGSEV	90.5	1.5	(49)		sp|Q8IZJ1|UNC5B_HUMAN. 458-462/31-34
LLQYWSQEL	87.9	1.5	(51)		
IVGAETFYV	93.9	1.6	(51)		
QMHEDVISL	93.9	1.6	(49)		
QLQARILAV	109.4	1.8	(51)		Q9P2M7|CING_HUMAN. 1138-1143/660-662
HLEGKIILV	150.6	2.3	(49)		
RMYSPISIL	162.9	2.3	(51)		Q9P225|DYH2_HUMAN. 1840-1843/3935-3939
HLEGKVILV	177.4	2.5	(51)		Q8N2C7|UNC80_HUMAN. 264-267/2799-2803
EMMTACQGV	210.4	2.9	(49)		
TLQEQIAWM	259.4	3.3	(49)		
FLQSRPEPT	371.6	4.1	(51)		
MTNNPPIPV	427.6	4.4	(50)		
QLTEVVQKI	424.7	4.4	(49)		

List of 9mer non-spliced peptides derived from various strains of HIV and predicted to bind HLA-A*02:01 complex with an IC_50_ ≤ 500 nM. These peptides also failed to trigger a specific CD8^+^ T cell response in HIV-infected donors (see Materials & Methods). The related papers are cited. The corresponding prediction of the peptide-HLA-A*02:01 binding affinity is reported as IC_50_ and rank, and it was computed by applying SMM algorithm. The potential human origin of the same sequences through peptide splicing by allowing either only cis-spliced peptides with intervening sequence ≤ 25 amino acid residues (cis-spliced 25, int. seq.) or any cis-spliced peptides is described through the UniprotKB's protein code and their location within the antigen.

### Modeling of Protein 3D Structures

For visualization purpose, the structures of Gag-Pol polyprotein of the HIV strain MVP5180 and of the human Major Vault protein (MVP) were predicted and visualized through the fully automated protein structure homology-modeling server, accessible via Expasy web server (52).

### Data Availability

A summary of the files accessible via repository is reported in the following Mendeley dataset: http://dx.doi.org/10.17632/hw686hytfs.1.

The mTEC's RNA sequencing data published by Pinto et al. (43) are available at Gene Expression Omnibus (GEO) under identifier GSE49625.

The single-cell RNA sequencing of TECs in human embryos published by Zeng et al. (44) are available at Gene Expression Omnibus (GEO) under identifier GSE133341.

## Results

### Estimation of the Upper Bond Frequency of Viral-Human *zwitter* Epitope Candidates

**Figure 2:**
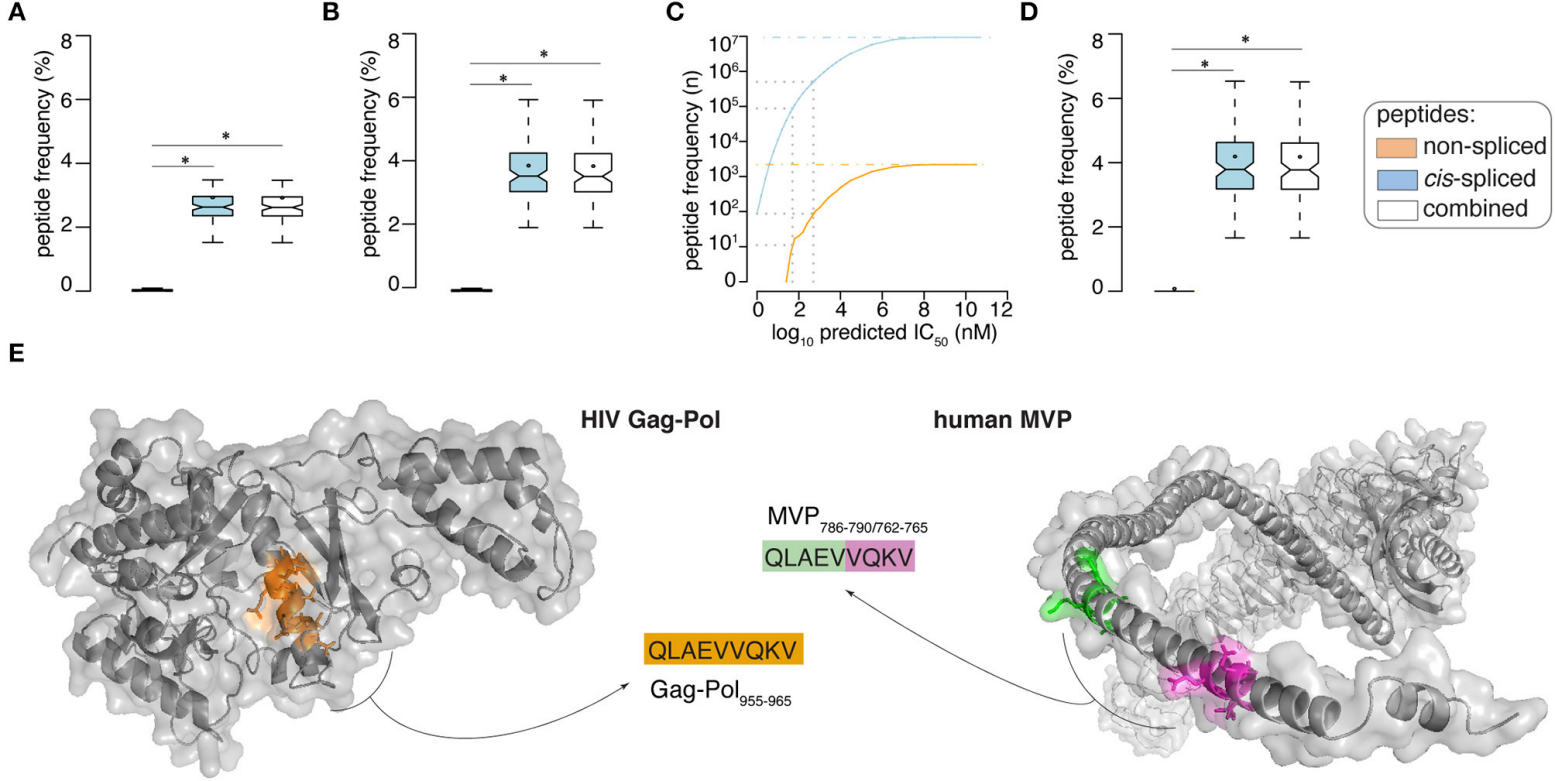
Viral-human *zwitter* epitope candidate frequency and examples. **(A,B)** Frequency of viral-human 9-mer **(A)** *zwitter* peptides and **(B)** HLA-A02:01-restricted (predicted IC_50_ ≤ 500 nM) *zwitter* epitope candidates, compared to their cognate viral peptide database and considering the whole human proteome database. **(C)** Number of viral-human *zwitter* non-spliced and *cis*-spliced epitope candidates depending on the peptide-HLA-A02:01predicted IC_50_. Gray dot lines mark the predicted IC_50_ of 500 nM and 50 nM. The blue and orange dot lines depict the number of viral-human *zwitter cis*-spliced and non-spliced peptide without peptide-HLA-A02:01predicted IC_50_ cut-off. **(D)** Frequency of HLA-A02:01-restricted (predicted IC_50_ ≤ 50 nM) viral-human *zwitter* 9mer epitope candidates, compared to their cognate viral peptide database and considering the whole human proteome database. **(E)** Example of HIV-human zwitter epitope candidate QLAEVVQKV, which may be derived from HIV Gag-Pol as non-spliced peptide (Gag-Pol_955−963_), and from the human MVP as *cis*-spliced peptide (MVP_786−790/762−765_). Both peptides are depicted in the cognate antigens. Color code corresponds to Figure 1A. In **(A,B,D)** Box plots depict the median and 25–75 percentiles of peptides per virus. Bars represent 5–95 percentiles. Dots represent the mean. Significant difference between groups is labeled with * (see Supplementary Table 1).

By applying the *in silico* pipeline described in Figure 1B and focusing on 9mer peptides, which represent the majority of non-spliced and *cis*-spliced peptides in HLA-I immunopeptidomes (28, 36, 38, 42), we identified 2,340 and 9,350,135 theoretical viral-human *zwitter* non-spliced and *cis*-spliced 9mer peptides, respectively (Supplementary Table 3). On average per virus, these represent 0.06 and 2.93% of the pool of virus non-spliced and *cis* spliced 9mer peptides, respectively (Figure 2A). We then predicted their binding affinity to the most predominant HLA-I allele in Caucasian population, i.e., HLA-A^*^02:01, and filtered out all peptides with predicted IC_50_ > 500 nM. This step removed ~96% of the peptides (on average, only ~5% of peptides per virus are left; see Supplementary Figure 1A). This left 87 and 504,209 viral-human *zwitter* non-spliced and *cis*-spliced 9mer epitope candidates in total, which correspond, on average per virus, to 0.05 and 3.84% of the pool of HLA-A^*^02:01-restricted viral non-spliced and *cis*-spliced 9mer peptides, respectively (Figure 2B). This frequency did not account for antigen processing via the APP pathway and assumed that each and every non-spliced and *cis*-spliced peptide that could be produced by proteasomes was indeed produced. Therefore, it represents the upper bond of viral-human *zwitter* 9mer epitope candidates. Interestingly, viral-human *zwitter* peptides were more often predicted to bind HLA-A^*^02:01 with an IC_50_ ≤ 500 nM than non-*zwitter* peptides (Supplementary Figure 1B).

When we loosen up the IC_50_ cut-off, the number of viral-human *zwitter* non-spliced and *cis*-spliced 9mer epitope candidates would increase (Figure 2C). To further investigate the theoretical frequency of viral-human *zwitter* non-spliced and *cis*-spliced 9mer epitope candidates among the potentially immunodominant epitopes, we focused on a more stringent IC_50_ cut-off of 50 nM. For instance, Platteel et al. (23) reported a correlation between the immunogenicity of *cis*-spliced epitope candidates, their predicted binding affinity to H2-K^b^ (IC_50_ ≤ 2 nM) and the measured *cis*-spliced peptide-H2-K^b^ complex stability in a mouse model of *Listeria monocytogenes* infection. While, Assarsson et al. (9) showed that all vaccinia immunodominant HLA-A^*^02:01-restricted non-spliced epitopes analyzed in their study on a transgenic mouse model had a measured peptide-HLA-A^*^02:01 IC_50_ ≤ 50 nM. With this latter IC_50_ cut-off, 11 non-spliced and 87,154 *cis*-spliced peptides were left among the viral-human *zwitter* epitope candidates, which correspond, on average per virus, to 0.06 and 4.19% of the pool of HLA-A^*^02:01-restricted (predicted IC_50_ ≤ 50 nM) viral non-spliced and *cis*-spliced 9mer peptides, respectively (Figure 2D).

### Example of T Cell Tolerance Against Viral-Human *zwitter* Epitope Candidate

As proof of principle, we selected a pool of HIV-derived HLA-A^*^02:01-restricted 9mer peptides, which were demonstrated to be non-immunogenic in previous studies (see Materials and Methods). Among them, we selected non-spliced peptides that were predicted to bind HLA-A^*^02:01 complex with IC_50_ ≤ 500 nM and, upon testing for CD8^+^ T cell response in HIV patients, were non-immunogenic (Table 1). We investigated whether any of them may also have been a viral-human *zwitter* 9mer epitope candidate. We considered both *cis*-spliced peptides with intervening sequence shorter than 26 amino acid residues, as in the rest of the study, as well as any theoretical *cis*-spliced peptide computed from the human proteasome. Out of twenty peptides with these characteristics, we identified the peptide QLAEVVQKV, which may derive from the Gag-Pol polyprotein of the HIV strain MVP5180 (Gag-Pol_955−963_). This epitope candidate has a predicted IC_50_ = 50 nM for HLA-A^*^02:01 (Table 1). Despite the good binding affinity, this epitope candidate did not trigger a PBMC response in HIV patients, according to Perez et al. (49). In their cohort of 31 HIV patients, 10 were HLA-A^*^02:01^+^ and none of them recognized the epitope candidate upon peptide stimulation. No other studies showed a recognition of this epitope candidate by CD8^+^ T cells, to our knowledge. According to our computation, the same peptide sequence may also derive from the Major Vault protein as a *cis*-spliced peptide—i.e., MVP_786−790/762−765_ [QLAE][VVQKV]—with intervening sequence smaller than 26 amino acid residues (Figure 2E). MVP's gene mRNA was identified in mTECs by both Pinto et al. (43) and Zeng et al. (44), thereby suggesting its expression in mTECs and, in theory, the potential presentation of the MVP_786−790/762−765_ *cis*-spliced epitope candidate to thymocytes. That might lead to negative selection of CD8^+^ T cell clones recognizing the peptide QLAEVVQKV, which might explain the absence of immunogenicity of the Gag-Pol_955−963_ [QLAEVVQKV] in HLA-A^*^02:01^+^ HIV patients.

If we expanded our research to any *cis*-spliced epitope candidate, regardless of the intervening sequence length, we identified six other *cis*-spliced epitope candidates with a sequence present in Table 1. Therefore, we should bear in mind that the pool of viral-human *zwitter* 9mer *cis*-spliced epitope candidates, which had an intervening sequence length smaller than 26 amino acid residues, represented only part of the whole theoretical *cis*-spliced peptides.

### Estimation of Viral-Human *zwitter* Epitope Candidate Frequency Weighing Up mTEC Transcriptome

Viral-human *zwitter* non-spliced and *cis*-spliced 9mer epitopes may impinge upon the functional CD8^+^ T cell repertoire through both central and peripheral tolerance. Herein, we focused solely on the negative selection step of the central tolerance. We hypothesized that TCRαβ T cell clones that recognize self-derived *zwitter* epitopes bound to HLA-I complexes of mTECs and other professional APCs with high avidity are tolerized.

In tolerance, the amount of antigen presented at the cell surface is relevant to the fate of T cell clones (5). Although gene expression does not mirror the HLA-I immunopeptidomes, it appears, to some extent, to be a predictor of antigen presentation (46). Bearing this in mind, we repeated our analysis by weighing up the probability of an antigen to be represented in mTEC's HLA-I immunopeptidome, based on transcriptome data from either microarray analysis of human mTECs (43) or single-cell RNA sequencing of TECs in human embryos (44).

To this end, we transformed gene expression values of mTECs into probabilities of antigens being represented in HLA-I immunopeptidomes through a crude model for antigen presentation based on the gene expression values (see Material and Methods). Furthermore, in our analysis, the probability of an antigen to be represented in mTEC's HLA-I immunopeptidomes was weighted by the number of *zwitter* non-spliced and *cis* spliced 9mer peptides predicted to bind HLA-A^*^02:01 (with IC_50_ ≤ 500 nM) and theoretically derived from that antigen (see Material and Methods). Indeed, the chance of an antigen being presented in HLA-I immunopeptidomes also depends on the number of HLA-I-binding peptides that could be derived from that given antigen. Since we introduced a probability score in our analysis, we had to sample the viral-human *zwitter* non-spliced and *cis*-spliced 9mer epitope candidate pool, thereby estimating the average frequency rather than the absolute frequency of these peptides, which has been shown so far.

**Figure 3:**
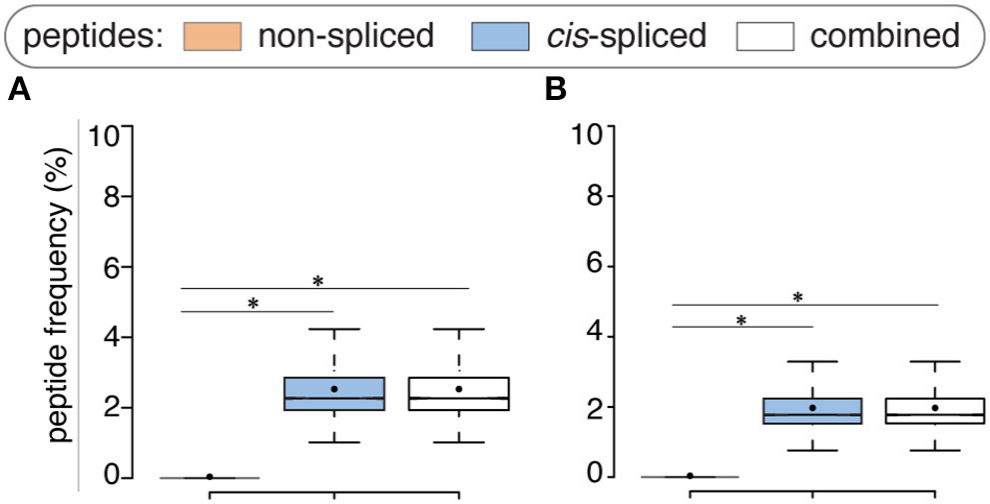
Viral-human *zwitter* epitope candidates considering mTEC's transcriptome. **(A)** Frequency of HLA-A02:01-restricted viral-human *zwitter* 9mer epitope candidates compared to their cognate viral peptide databases considering the human mTEC transcriptome computed either **(A)** from (43) or **(B)** from (44). Box plots depict the median and 25–75 percentiles. Bars represent 5–95 percentiles. Dots represent the mean. Significant difference between groups is labeled with * (see Supplementary Table 1).

Compared to the whole human proteome, incorporation of potential antigen repertoire based on mTEC transcriptome resulted in a decreased average number of both *zwitter* non-spliced and *cis*-spliced epitope candidates. On average per virus, 0.04% and 2.53% of the pool of HLA-A^*^02:01-restricted virus non-spliced and *cis*-spliced 9mer epitope candidates, respectively, were *zwitter* peptides using Pinto's RNA sequencing database (Figure 3A). Similar results were obtained using Zeng's RNA sequencing database (Figure 3B).

### Estimation of Viral-Human *zwitter* Epitope Candidate Frequency Weighing Up *cis*-PCPS Frequency

The computation done so far did not take into account the frequency of peptides produced by proteasomes through peptide hydrolysis and peptide *cis*-splicing and presented at the cell surface. Despite not being physiological, one of the most detailed approaches to determine what proteasomes can produce via peptide hydrolysis and peptide splicing is, in our experience, the measurement through MS of non-spliced and *cis*-spliced peptides produced *in vitro* by purified 20S proteasomes during the degradation of synthetic polypeptides recapitulating antigenic sequence. Correspondence between *in vitro* experiments carried out with purified 20S proteasomes and *in cellulo* and *in vivo* experiments has been demonstrated in various studies investigating both viral and tumor epitopes (23, 24, 26, 27, 30, 53–62). The analysis of *in vitro* digestions of synthetic polypeptides by 20S proteasomes showed that, although these proteases can cleave—and likely ligate—any amino acid, they have substrate sequence preferences (34). It also showed that *cis-*spliced peptides are produced, on average, in significantly smaller amount than non-spliced peptides by proteasomes (26, 32, 33). Therefore, not all non-spliced peptides, and even less *cis*-spliced peptides, are likely generated by proteasomes in sufficient amount to be detected *in vitro* by MS as well as to survive all steps of HLA-I APP pathway.

We weighed up the impact of this phenomenon in our computational analysis by gathering information from two experimental dataset sources measured by MS: a large database of non-spliced and spliced peptides produced *in vitro* by purified proteasomes (34) and HLA-I immunopeptidome elutions.

Through the analysis of *in vitro* digestion database (34), we estimated that ~27% of all theoretical non-spliced 9mer peptides that could be produced by proteasomes are in fact generated in a detectable amount. This figure is much smaller for *cis*-spliced peptides.

The frequency of *cis*-spliced peptides in HLA-I immunopeptidomes is still a controversial topic, with their frequency in HLA-I immunopeptidomes being estimated in a range from 1 to 34%, depending on the method used for their identification (31).

Using these two sets of information, we determined the relative frequency of non-spliced and *cis* spliced peptides generated by proteasomes and presented in HLA-I immunopeptidomes compared to all theoretical non-spliced and *cis*-spliced peptide products; we then implemented it into our model to better estimate viral-human *zwitter* peptide frequency. Based on this new analysis, we randomly selected non-spliced and *cis*-spliced peptides from our viral and human proteome databases, repeated sampling 600 times to reach statistical power and then repeated our entire analysis for each sample (Figure 1C).

**Figure 4:**
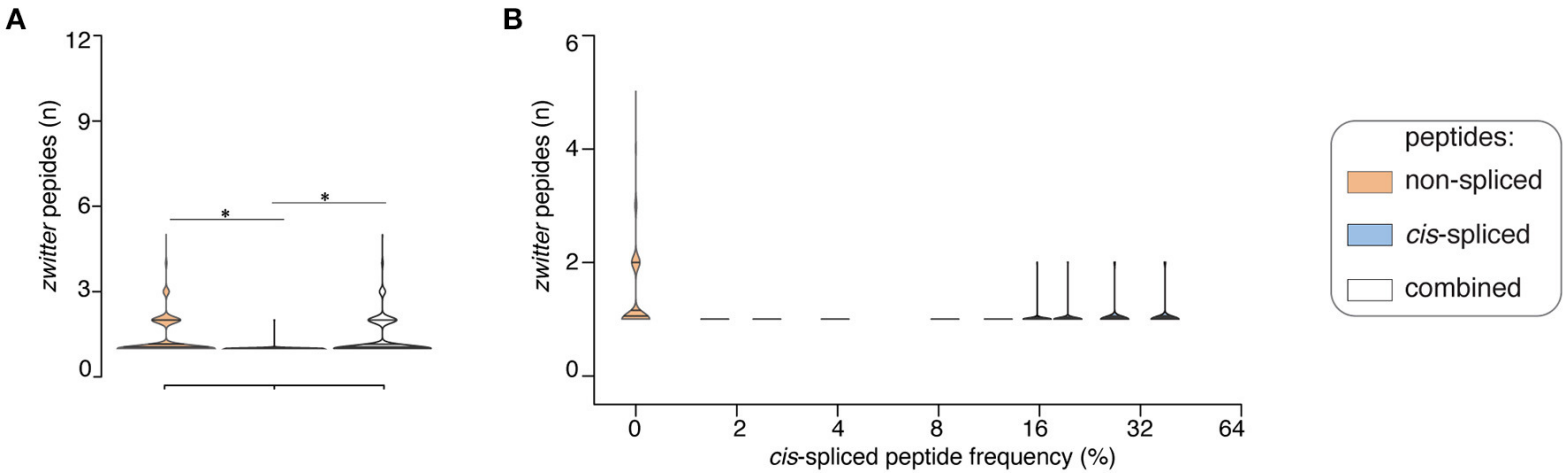
Viral-human *zwitter* epitope candidates considering *cis-*spliced peptide frequency in HLA-I immunopeptidomes. **(A)** Distribution of the number of viral-human 9mer HLA-A02:01-restricted (non-spliced, *cis*-spliced and combined) *zwitter* epitope candidates per virus across all 600 random samples presented as violin plots (rotated densities). Significant difference between groups is labeled with * (see Supplementary Table 1). This analysis was carried out by hypothesizing that *cis*-spliced peptides represent ~15% of peptides in HLA-I immunopeptidomes and by using the whole human proteome as database. The distribution of the number of viral-human *zwitter cis*-spliced epitope candidates has been displayed among viruses that had at least one *zwitter* peptide. **(B)** Number of HLA-A*02:01-restricted viral-human *zwitter* non-spliced and *cis*-spliced 9mer epitope candidates per virus per sampling iteration, depending on a broad range of theoretical *cis*-spliced peptide frequencies in HLA-I immunopeptidomes. Here, viral proteomes are compared to the whole human proteome database. The number of viral-human *zwitter* non-spliced and *cis*-spliced 9mer epitope candidates per virus per iteration has been computed among viruses that had at least one *zwitter* peptide.

If we assumed a ~15% *cis*-spliced peptide frequency in HLA-I immunopeptidomes, over all randomly sampled peptide pools, we identified, on average, a total of 7 HLA-A^*^02:01-restricted viral-human *zwitter* non-spliced 9mer epitope candidates. They correspond to 0.079% of the pool of HLA-A^*^02:01-restricted virus non-spliced 9mer peptides. This figure strongly varied from virus to virus. On average of sampling, 6 viruses had at least one HLA-A^*^02:01-restricted viral-human *zwitter* non-spliced 9mer peptide. No more than 5 epitope candidates per virus were estimated in this analysis (Figure 4A). In the same analysis, we identified, on average, a total of 0.3 HLA-A^*^02:01-restricted viral-human *zwitter cis*-spliced 9mer epitope candidates. They correspond to 0.0008% of the pool of HLA-A^*^02:01-restricted virus *cis*-spliced 9mer peptides, which is a frequency dramatically smaller than the 3.84% computed without accounting for *cis*-spliced peptide frequency in HLA-I immunopeptidomes (see Figure 2B). On average of sampling, only 1 virus had an HLA-A^*^02:01-restricted viral-human *zwitter cis*-spliced 9mer epitope candidate and no more than 2 epitope candidates per virus were estimated (Figure 4A).

Since *cis*-spliced peptide frequencies in HLA-I immunopeptidomes is so controversial, we repeated the non-spliced and *cis*-spliced peptides' sampling and downstream analysis considering a broad range of frequencies of *cis*-spliced peptides in HLA-I immunopeptidomes. As shown in Figure 4B, the overall picture did not change much. The average number of HLA-A^*^02:01-restricted viral-human *zwitter* non-spliced epitope candidates was estimated to be always largely higher than *cis*-spliced epitope candidates. Only few outliers of *cis*-spliced epitope candidates were identified when we assumed very large frequencies of *cis*-spliced peptide in HLA-I immunopeptidomes.

**Figure 5:**
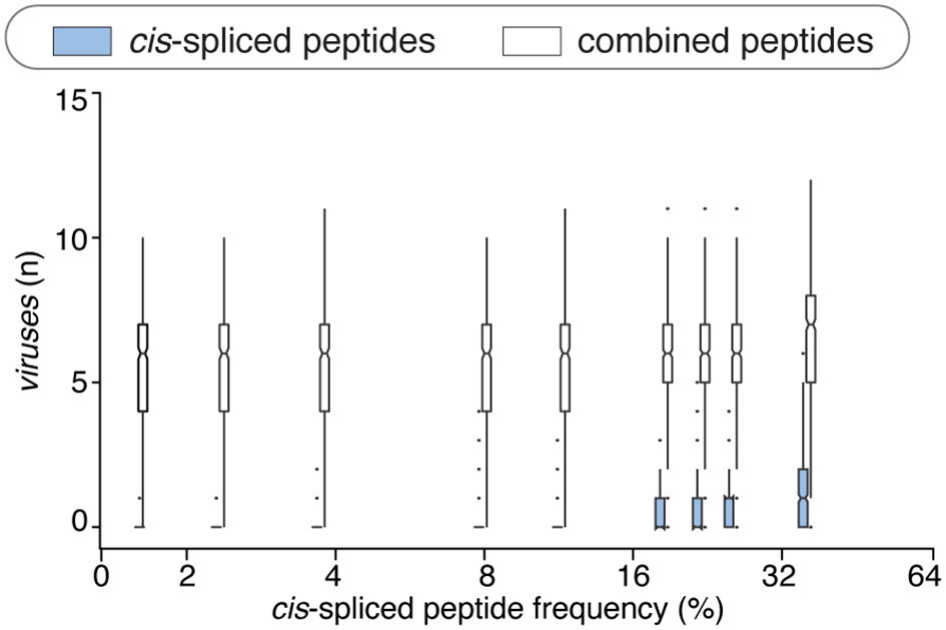
Viruses that have *zwittter* epitope candidates depending on *cis*-spliced peptide frequency in HLA-I immunopeptidomes. Average number of viruses that contain at least one HLA-A02:01-restricted viral-human *zwitter* 9mer peptide per iteration, depending on a broad range of theoretical *cis*-spliced peptide frequencies in HLA-I immunopeptidomes. Viral proteomes are compared to the whole human proteome database. The boxplots of the combined peptides have been slightly shifted on the x axis for representation purpose.

This phenomenon was reflected also in terms of number of viruses that, on average of sampling, had one or more HLA-A^*^02:01-restricted viral-human *zwitter* epitope candidates. The average number of viruses with one or more HLA-A^*^02:01-restricted viral-human *zwitter* epitope candidates was only increased by including cis-spliced epitope candidates if we assumed a frequency of *cis*-spliced peptides in HLA-I immunopeptidomes larger than 30% (Figure 5).

**Figure 6:**
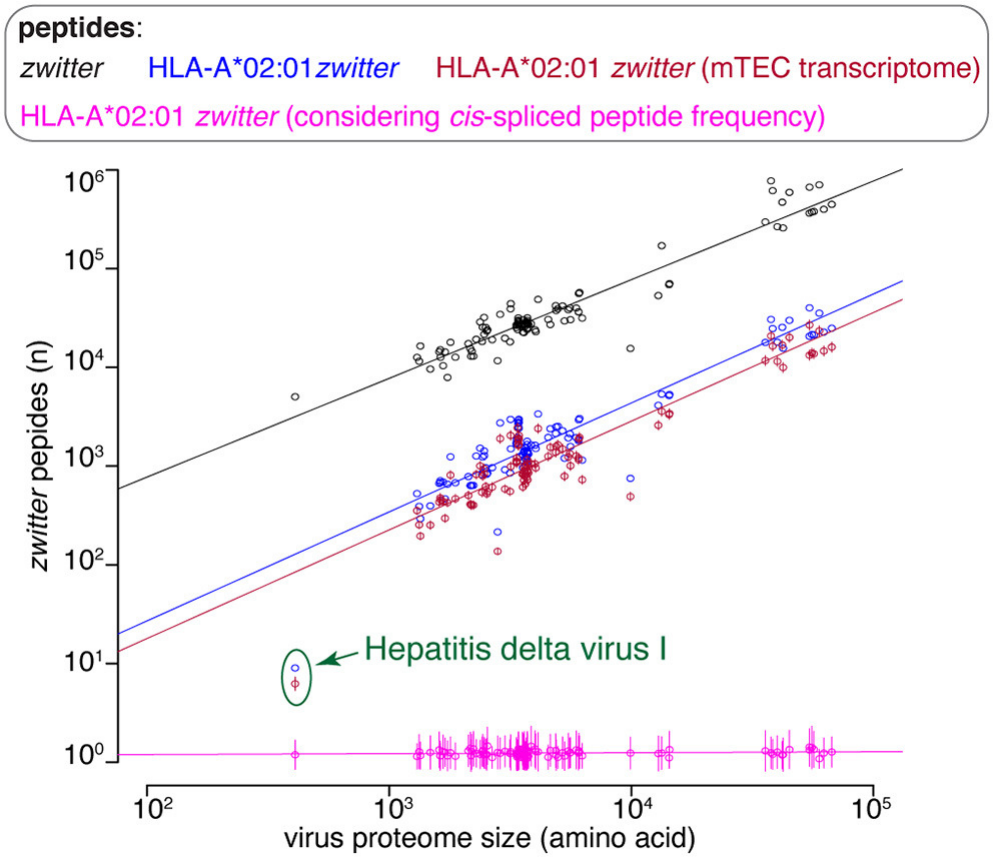
Viral-human *zwitter* epitope candidate frequency depends on virus length and sequence motifs. Number of viral-human *zwitter* combined (i.e., non-spliced + *cis* spliced peptides) 9mer peptides per virus, depending on the number of amino acid residues in its proteome. For the groups labeled in pink, we considered a *cis*-spliced peptide frequency of ~15%, as in Figure 3A. Viral-human *zwitter* 9mer peptides and HLA-A*02:01-restricted viral-human *zwitter* 9mer epitope candidates are represented with a dot each virus. HLA-A*02:01-restricted viral-human *zwitter* 9mer epitope candidates either using mTEC's RNA-based proteome database (43) or considering the theoretical *cis*-spliced peptide frequency in HLA-I immunopeptidomes are represented with a dot (mean) and bars (SD) of sampling iterations. Regression lines are shown. The Hepatitis delta virus I has an underrepresented number of HLA-A*02:01-restricted viral-human *zwitter* 9mer epitope candidates, which are here labeled.

There are various factors that can impinge upon the number of viral-human *zwitter* epitope candidates that could be derived from a given virus. One of them is the number of amino acid residues present in viral proteomes. The direct correlation between viral-human *zwitter* epitope candidates and the size of virus proteome databases was however stronger if we did not consider the frequencies of *cis*-spliced peptide in HLA-I immunopeptidomes (Figure 5, Supplementary Table 1). Another factor can be the sequence motifs of viral proteome, which may not favor the presentation of viral-human *zwitter* epitope candidates through a specific HLA-I allele. For example, this is the case of the Hepatitis delta virus I, which has an underrepresentation of viral-human *zwitter* epitope candidates among those that are predicted to bind HLA-A^*^02:01 molecules as compared to the total number of its theoretical viral-human *zwitter* peptides (Figure 6).

## Discussion

Despite proteasome-generated spliced epitopes being known about for more than a decade (61, 63), the potential implications of their presentation by HLA-I complexes only started to concern the scientific community in recent years when we and others showed that spliced peptides represented a sizeable portion of HLA-I immunopeptidomes (28, 36, 38, 42). One of these concerns was the hypothetical impact of spliced peptides on central and peripheral tolerance and on the repertoire of CD8^+^ T cells recognizing viruses. Indeed, the theoretical substantial sequence variability of *cis*-spliced peptides may strongly increase the number of viral-human *zwitter* epitope candidates, thereby reducing the ability of the CD8^+^ T cell repertoire able to recognize viruses (7, 64). Here we showed *in silico* evidence that *cis*-spliced peptides might not play such an unsettling role in the central and peripheral tolerance of the CD8^+^ T cell repertoire. The main reason is that *cis*-spliced peptides produced and presented through APP pathway represent just a tiny fraction of all theoretical *cis*-spliced peptide sequences, as suggested by biochemical and immunopeptidomics studies. According to our preliminary estimations, *zwitter cis*-spliced epitopes would only significantly impinge upon the virus-specific repertoire of CD8^+^ T cells if we assumed a very large frequency of these unconventional peptides in HLA-I immunopeptidomes. Although, our analysis was restricted to *cis*-spliced epitope candidates with intervening sequence shorter than 26 amino acid residues, which may represent only part of HLA-I spliced immunopeptidomes (36).

Additionally, we should bear in mind that our analysis did not consider two potentially important factors: CD8^+^ TCR specificity degeneracy and driving forces that can restrict the variety of non-spliced and *cis*-spliced peptides produced by proteasomes.

The former has already been investigated in a seminal work of Calis et al. (17), who focused on non-spliced epitope candidates. Some examples of TCR cross-recognition of pathogen-derived *cis*-spliced and non-spliced epitopes have been already reported (24, 25). However, we think that we would need data on a larger pool of TCRs before accounting for this factor in our model. To note, this aspect would be even more relevant if we wanted to extend this investigation to CD4^+^ T cell repertoire, bearing in mind that CD4^+^ TCR degeneracy is more pronounced than in CD8^+^ T cells, and *trans*-spliced peptides are under the spotlight in type 1 Diabetes (65–68).

The latter factor is the impact that substrate sequences have on both peptide hydrolysis and splicing. Proteasomes can cleave and likely splice after any amino acid, as confirmed by a large database of non-spliced and spliced peptides produced *in vitro* by these enzymes (34). However, peptide sequence motifs seem to impinge upon proteasome dynamics (69) as well as the variety and quantity of non-spliced and *cis*-spliced peptides that they generate (26, 33, 34, 70–72). This factor may reduce the variety of non-spliced and *cis*-spliced peptides that are finally presented through HLA-I complexes to CD8^+^ T cells, and thus alter the frequency of viral-human *zwitter* epitope candidates.

Finally, in future studies we might also consider the impact that proteasome isoforms might have on the frequency of *zwitter* epitope candidates. Indeed, standard proteasomes, immunoproteasomes and thymoproteasomes seem to have, at least from a quantitative perspective, different dynamics and substrate sequence preferences for both peptide hydrolysis and splicing (27, 33, 55, 56, 59, 69, 70, 73–75). This can impinge upon the proteome and antigenic landscape of both professional APCs and infected cells (28, 76), and ultimately upon central and peripheral tolerance of CD8^+^ T cells potentially specific for viral-human *zwitter* epitopes.

## Data Availability Statement

The datasets presented in this study can be found in online repositories. The names of the repository/repositories and accession number(s) can be found below: A summary of the files accessible via repository is reported in the following Mendeley dataset: http://dx.doi.org/10.17632/hw686hytfs.1. The mTEC's RNA sequencing data published by Pinto et al. (43) are available at Gene Expression Omnibus (GEO) under identifier GSE49625. The single-cell RNA sequencing of TECs in human embryos published by Zeng et al. (44) are available at Gene Expression Omnibus (GEO) under identifier GSE133341.

## Author Contributions

JL conceived the project. AM implemented the *in silico* pipelines and carried out the data analysis and figure preparation, which were supervised by JL and MM. CRRB identified the epitope candidates reported in Table 1. MM critically revised the immunological implication of the study and the project development. MM, JL, AM, and CRRB wrote the manuscript. All authors contributed to the article and approved the submitted version.

## Conflict of Interest

The authors declare that the research was conducted in the absence of any commercial or financial relationships that could be construed as a potential conflict of interest.
