# Supplementary material for: Proteasome-Generated cis-Spliced Peptides and Their Potential Role in CD8+ T Cell Tolerance
Source: Front Immunol. 2021 Feb 24;12:614276. doi: 10.3389/fimmu.2021.614276 (PMC7943738; doi:10.3389/fimmu.2021.614276)
Supplement: Supplementary file 1 [file Data_Sheet_1.pdf]

Supplementary information

Proteasome-generated *cis*-spliced peptides and their potential role in CD8^{+} T cell tolerance

$ Correspondence to: michele.mishto@kcl.ac.uk, jliepe@mpibpc.mpg.de.

Table S1	Statistical test values
Table S2	List of virus strains included in the study
Table S3	List of viral-human *zwitter* peptides
Figure S1	Frequency of viral-human *zwitter* non-spliced and *cis*-spliced peptides predicted to bind HLA-A*02:01 complex

Kolmogorov-Smirnov test (difference between groups)			
**Figure**	**Group A**	**Group B**	**p-value**
2A	*Zwitter* non-spliced peptides	*Zwitter cis*-spliced peptides	2.2e-16
2A	*Zwitter* non-spliced peptides	*Zwitter* non-spliced and *cis*-spliced peptides (combined *zwitter* peptides)	2.2e-16
2A	*Zwitter cis*-spliced peptides	*Zwitter* non-spliced and *cis*-spliced peptides (combined *zwitter* peptides)	1
2B	*Zwitter* non-spliced peptides	*Zwitter cis*-spliced peptides	2.2e-16
2B	*Zwitter* non-spliced peptides	*Zwitter* non-spliced and *cis*-spliced peptides (combined *zwitter* peptides)	2.2e-16
2B	*Zwitter cis*-spliced peptides	*Zwitter* non-spliced and *cis*-spliced peptides (combined *zwitter* peptides)	1
2D	*Zwitter* non-spliced peptides	*Zwitter cis*-spliced peptides	2.2e-16
2D	*Zwitter* non-spliced peptides	*Zwitter* non-spliced and *cis*-spliced peptides (combined *zwitter* peptides)	2.2e-16
2D	*Zwitter cis*-spliced peptides	*Zwitter* non-spliced and *cis*-spliced peptides (combined *zwitter* peptides)	1
3A	*Zwitter* non-spliced peptides	*Zwitter cis*-spliced peptides	2.2e-16
3A	*Zwitter* non-spliced peptides	*Zwitter* non-spliced and *cis*-spliced peptides (combined *zwitter* peptides)	2.2e-16
3A	*Zwitter cis*-spliced peptides	*Zwitter* non-spliced and *cis*-spliced peptides (combined *zwitter* peptides)	1
3B	*Zwitter* non-spliced peptides	*Zwitter cis* spliced peptides	2.2e-16
3B	*Zwitter* non-spliced peptides	*Zwitter* non-spliced and *cis*-spliced peptides (combined *zwitter* peptides)	2.2e-16
3B	*Zwitter cis*-spliced peptides	*Zwitter* non-spliced and *cis*-spliced peptides (combined *zwitter* peptides)	1
4A	*Zwitter* non-spliced peptides	*Zwitter cis* spliced peptides	1.992e-11
4A	*Zwitter* non-spliced peptides	*Zwitter* non-spliced and *cis*-spliced peptides (combined *zwitter* peptides)	1
4A	*Zwitter cis*-spliced peptides	*Zwitter* non-spliced and *cis*-spliced peptides (combined *zwitter* peptides)	2.22e-11

**Correlation coefficients between virus length and number of different types of *zwitter* peptides**		
**Type of *zwitter* peptide**	**Pearson Correlation Coefficient (C)**	**p-value**
zwitter peptides	0.904	2.2e16
HLA-A*02:01-restricted *zwitter* peptides	0.943	2.2e-16
HLA-A*02:01-restricted *zwitter* peptides based on RNA-based proteome database [1]	0.955	2.2e-16
HLA-A*02:01-restricted *zwitter* peptides assuming ~15% frequency of *cis*-spliced peptides in HLA-I immunopeptidomes	0.172	7,40e-02

Table S1. Statistical test values.

Table S2. List of virus strains included in the study. All viruses included in this study are listed with their respective strains. The list is available as .csv file.

Table S3. List of viral-human *zwitter* peptides. List of viral-human *zwitter* 9mer peptides and HLA-A*02:01-binder *zwitter* 9mer peptides. The table columns indicate viral sequence, human sequence (identical to viral sequence), predicted peptide-HLA-A*02:01 binding affinity (IC_{50} in nM), origin of viral and human sequences, type of viral and human sequences. Origins are indicated as ID_p1 _p4 and ID_p1_p2_p3_p4 for non-spliced and *cis*-spliced peptides, respectively, where ID is the antigens Uniprot ID, p1 is the start position of the peptide in the antigen, p4 is the end position of the peptide in the antigen, and p2 and p3 are the splice sites of the *cis*-spliced peptide. The list is available as .csv file in the Mendeley repository.

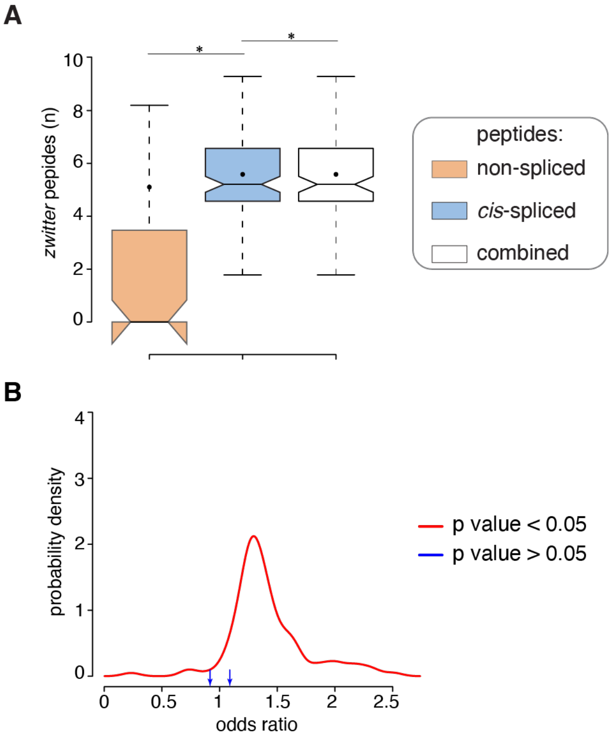

Figure S1. Frequency of viral-human *zwitter* non-spliced and *cis*-spliced peptides predicted to bind HLA-A*02:01 complex. (**A**) Frequency of viral-human *zwitter* non-spliced and *cis*-spliced peptides predicted to bind HLA-A*02:01 complex as compared to all potential viral non-spliced and *cis*-spliced peptides**.** Prediction was carried out by applying SMM Method [2] and an IC_{50} cut-off of 500 nM. (**B**) Odds ratios of viral-human *zwitter* vs non-*zwitter* 9mer peptides predicted to bind HLA-A*02:01 complexes**.** For all tested viruses, except Human Papilloma virus type 1 and Human Parvovirus B19, odds ratios tested significant and their distribution is shown in red. Odds ratios for non-significant viruses are indicated by the two blue arrows.

References

[1] S. Pinto, C. Michel, H. Schmidt-Glenewinkel, N. Harder, K. Rohr, S. Wild, B. Brors, and B. Kyewski, Overlapping gene coexpression patterns in human medullary thymic epithelial cells generate self-antigen diversity. Proc Natl Acad Sci U S A 110 (2013) E3497-505.

[2] B. Peters, Tong, W., Sidney, J., Sette, A., Weng, Z, Examining the independent binding assumption for binding of peptide epitopes to MHC-I molecules, Bioinformatics 2003, pp. 1765-1772.
